# Endoplasmic Reticulum Stress-Mediated Activation of p38 MAPK, Caspase-2 and Caspase-8 Leads to Abrin-Induced Apoptosis

**DOI:** 10.1371/journal.pone.0092586

**Published:** 2014-03-24

**Authors:** Ritu Mishra, Anjali A. Karande

**Affiliations:** Department of Biochemistry, Indian Institute of Science, Bangalore, Karnataka, India; University of Hong Kong, Hong Kong

## Abstract

Abrin from *Abrus precatorius* plant is a potent protein synthesis inhibitor and induces apoptosis in cells. However, the relationship between inhibition of protein synthesis and apoptosis is not well understood. Inhibition of protein synthesis by abrin can lead to accumulation of unfolded protein in the endoplasmic reticulum causing ER stress. The observation of phosphorylation of eukaryotic initiation factor 2α and upregulation of CHOP (CAAT/enhancer binding protein (C/EBP) homologous protein), important players involved in ER stress signaling by abrin, suggested activation of ER stress in the cells. ER stress is also known to induce apoptosis via stress kinases such as p38 MAPK and JNK. Activation of both the pathways was observed upon abrin treatment and found to be upstream of the activation of caspases. Moreover, abrin-induced apoptosis was found to be dependent on p38 MAPK but not JNK. We also observed that abrin induced the activation of caspase-2 and caspase-8 and triggered Bid cleavage leading to mitochondrial membrane potential loss and thus connecting the signaling events from ER stress to mitochondrial death machinery.

## Introduction

Abrin, obtained from the mature seeds of *Abrus precatorius* plant is a member of the type II ribosome inactivating protein (RIP) family and is a potent toxin [1], [2]. It is composed of two polypeptide chains, an enzymatic A chain that has RNA-N-glycosidase activity and a galactose-specific lectin, the B chain, that facilitates the entry of the toxin in cells [3]. After entering cells, a few molecules of abrin reach the endoplamic reticulum (ER) via the retrograde transport, where the disulfide bond between the A and the B subunits gets cleaved. Then the A chain escapes into the cytosol where it binds to its target, the α-sarcin loop of the 28S ribosomal RNA and inhibits protein synthesis [4]. Apart from inhibition of protein synthesis, exposure of cells to abrin leads to the loss of mitochondrial membrane potential (MMP) resulting in the activation of caspases and finally apoptosis [4], [5]. However, whether apoptosis is dependent on the inhibition of protein synthesis is not elucidated. Inhibition of protein synthesis by the catalytic A subunit of abrin could result in accumulation of unfolded proteins in the ER leading to ER stress and triggering the unfolded protein response (UPR) pathway. The ER resident trans-membrane sensors IRE1 (Inositol-requiring enzyme 1), PERK (PKR-like ER kinase) and ATF6 (Activating transcription factor 6) are the major effectors of UPR in mammalian cells [6], [7]. These sensors increase the levels of chaperones and inhibit translation to restore protein homeostasis. However, if the ER stress is prolonged, apoptotic pathways get activated to remove severely damaged cells in which protein folding defects cannot be resolved [8], [9].

Recent studies have shown that ER stress-induced apoptosis can activate initiator caspases such as caspase-2 [10], [11], [12] and caspase-8 [13], [14], [15] which eventually lead to the mitochondrial membrane potential loss and activation of downstream effectors capases-9 and -3 [16], [17]. Furthermore, when ER stress is extensive, UPR induces activation of IRE1/ASK1/JNK [18], [19], [20] and also the p38 MAPK pathway which leads to apoptosis [21]. Abrin-triggered cell death via the mitochondrial pathway was first demonstrated in our laboratory on Jurkat cells [6]. Therefore, we initiated investigations on the role of caspase-2, caspase-8 and stress kinases in abrin-induced apoptosis in the same cell line.

RIPs such as Shiga toxin have been shown to induce direct DNA damage [22] and activate p53/ATM-dependent signaling pathway in mammalian cells [23]. Studies were also performed to investigate whether abrin can induce direct DNA damage.

## Results

### Inhibition of Protein Synthesis and Apoptosis by Abrin

**Figure 1:**
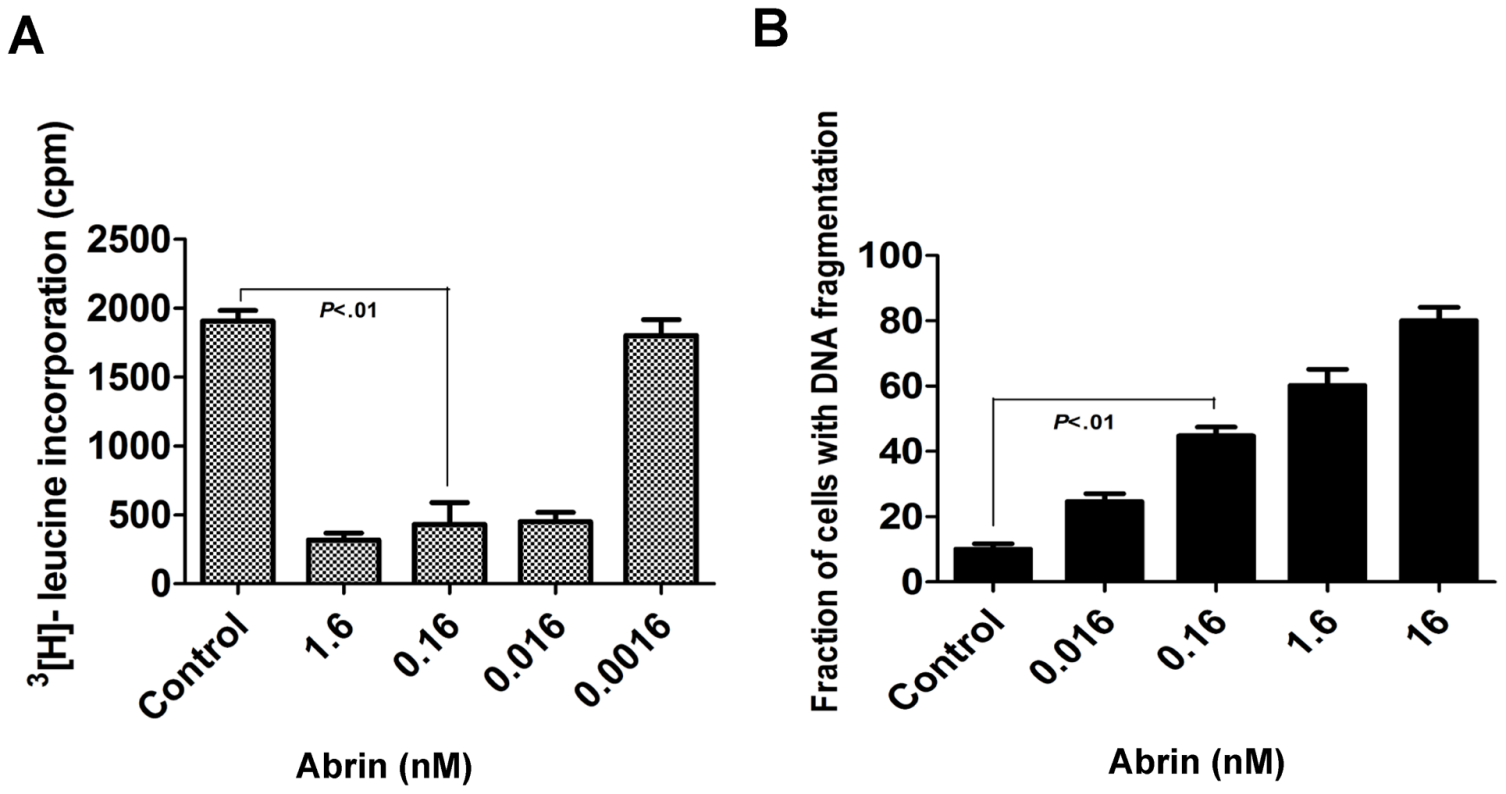
Abrin mediated protein synthesis inhibition and apoptosis in Jurkat cells. (A) Jurkat cells were treated with different concentrations of abrin for 8 h and protein synthesis was measured by incorporation of [^3^H]-leucine. (B) Jurkat cells were treated with varying concentrations (16 nM – 0.016 nM) of abrin for 12 h. After the treatments cells were harvested, fixed with 70% ethanol, stained with propidium iodide and quantified for apoptotic population. Each bar is presented as mean ± SE of triplicate samples.

Inhibition of protein synthesis was studied in Jurkat cells after 8 h of abrin treatment. Figure 1A shows the dose dependent inhibition of protein synthesis mediated by abrin. We observed significant inhibition of translation with a concentration as low as 0.016 nM (1 ng/ml) (Figure 1A). We also checked apoptosis induced by varying concentration of abrin ranging from 16 nM (1 μg/ml) to 0.016 nM (1 ng/ml) for 10 h using propidium iodide. Using flow cytometry abrin was shown to induce apoptosis in cells in a dose-dependent manner as quantified by the percentage of the sub G0/G1 cell population. Double staining with Annexin-V-FITC and propidium iodide was also carried out to confirm that cells die of apoptosis and not necrosis as shown in Figure S2. A high proportion of Annexin-V-FITC positive/PI negative cells were observed in each treatment, indicating the prevalence of apoptosis versus necrosis. A concentration of 0.16 nM of abrin showed considerable apoptosis in 10 h, hence this was chosen for all further studies (Figure 1B).

### Involvement of ER Stress in Abrin-mediated Apoptosis

**Figure 2:**
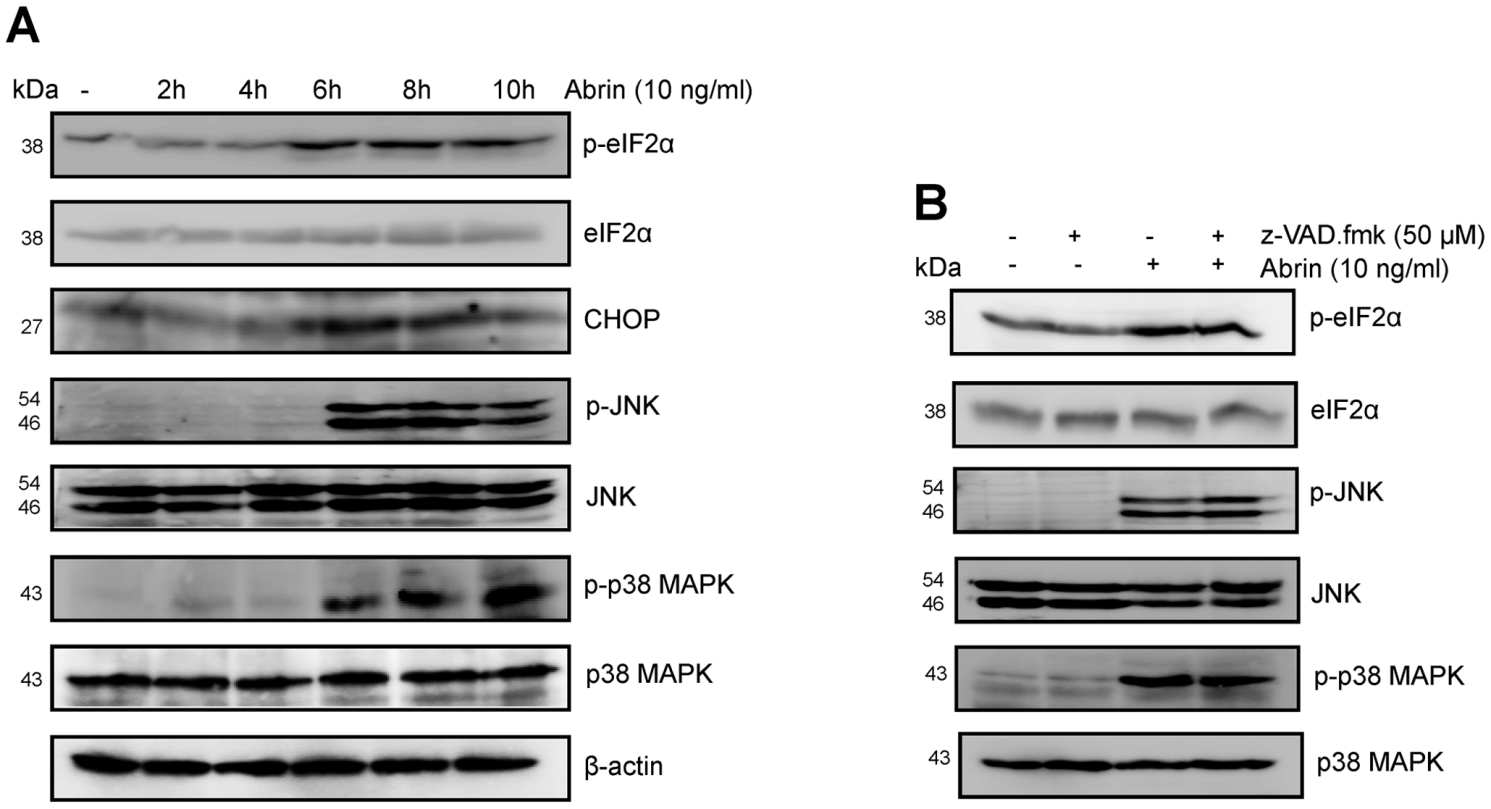
Abrin induces ER stress in Jurkat cells. (A) After the treatment of Jurkat cells with abrin (10 ng/ml) for different time intervals (0–10 h), whole cell lysates were prepared and analysed by Western blot for the total and phosphorylated levels of eIF2α, JNK and p38 MAPK using specific antibodies (B) Jurkat cells were pretreated with 50 μM z-VAD.fmk for 2 h followed by abrin (10 ng/ml) for 10 h. After the treatments, whole cell lysates were prepared and analysed by Western blot for the total and phosphorylated level of eIF2α, JNK and p38 MAPK using specific antibodies.

Activation of ER stress has been demonstrated in many cell lines treated with type II RIPs [24], [25]. Therefore, we explored whether abrin induces ER stress in Jurkat cells. As shown in Figure 2A, treatment with 10 ng/ml abrin significantly increased phosphorylation of eIF2α (eukaryotic initiation factor 2α) and expression of CHOP that are markers for ER stress, by 6 h. ER stress is also known to induce the phosphorylation of JNK (c-Jun N-terminal kinase) and p38 MAPK (p38 mitogen-activated protein kinase) [18], [19], [20] which in turn can lead to upregulation of several transcription factors like ATF2 and CHOP. After 6 h of abrin treatment, the phosphorylation of JNK and p38 MAPK was observed to increase significantly without a change in the level of total JNK and p38 MAPK proteins (Figure 2A). Pretreatment of cells with broad spectrum pan-caspase inhibitor (z-VAD.fmk) failed to block the phosphorylation of eIF2α, JNK and p38 MAPK, suggesting that the activation of ER stress is upstream of mitochondrial cytochrome-c release and therefore upstream of the apoptotic caspase cascade (Figure 2B).

### Abrin Induces Caspase-2 and Caspase-8 Activation

**Figure 3:**
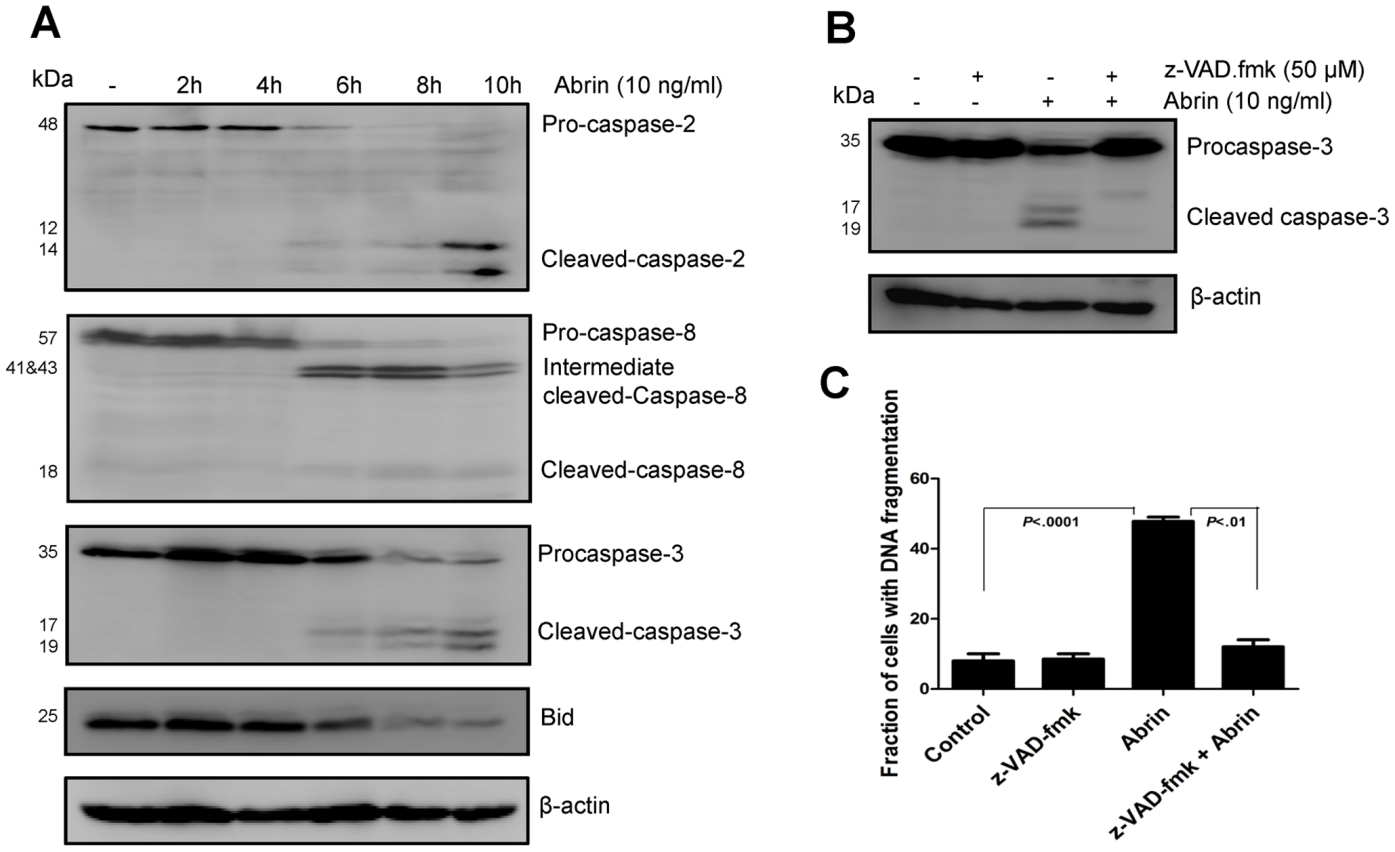
Abrin-induced apoptosis activates caspase -2, -8 and -3. (A) Jurkat cells were treated with abrin (10 ng/ml) for different time intervals (0–10 h). After the treatment, whole cell lysates were prepared and analysed by Western blot for cleaved caspase-2, -8, -3 and Bid using specific antibodies. Equal protein loading was checked by stripping and re-probing the membranes for β-actin. (B) Jurkat cells were pretreated with 50 μM z-VAD.fmk for 2 h followed by abrin (10 ng/ml) for 10 h. After the treatments whole cell lysates were prepared and analysed by Western blot for cleaved caspase-3 using specific antibody. Equal protein loading was checked by stripping and re-probing the membranes for β-actin. (C) After similar treatments with 50 μM z-VAD.fmk and abrin, cells were harvested, fixed with 70% ethanol, stained with propidium iodide and quantified for apoptotic population. Each bar is presented as mean ± SE of triplicate samples.

Several reports have suggested the involvement of initiator caspase-2 and -8 in ER stress induced apoptosis [10], [11], [12], [13], [14], [15]. Activation of these caspases by abrin was investigated at early time points (2–10 h) in Jurkat cells. Upon treatment with abrin, prominent cleavage of both caspase-2 and -8 was seen after 6 h of treatment (Figure 3A). Since caspase-2 and -8 are known to cleave the proapoptotic protein Bid, we also analysed the status of Bid in the cells. Bid cleavage was assessed as decrease in the levels of full length protein, which became apparent from 6 h of treatment (Figure 3A). Cleavage of caspase-3 was also observed by 6 h of treatment. The role of caspases in abrin-induced apoptosis was confirmed by pretreating cells with 50 μM broad spectrum pan-caspase inhibitor (z-VAD.fmk). Abrin mediated apoptosis and caspase-3 cleavage was significantly decreased in the presence of the inhibitor (Figure 3B & 3C). These results suggested that abrin-induced apoptosis in Jurkat cells involves caspase-dependent mechanism.

### Role of Caspase-2 and Caspase-8 in Abrin-induced Apoptosis

**Figure 4:**
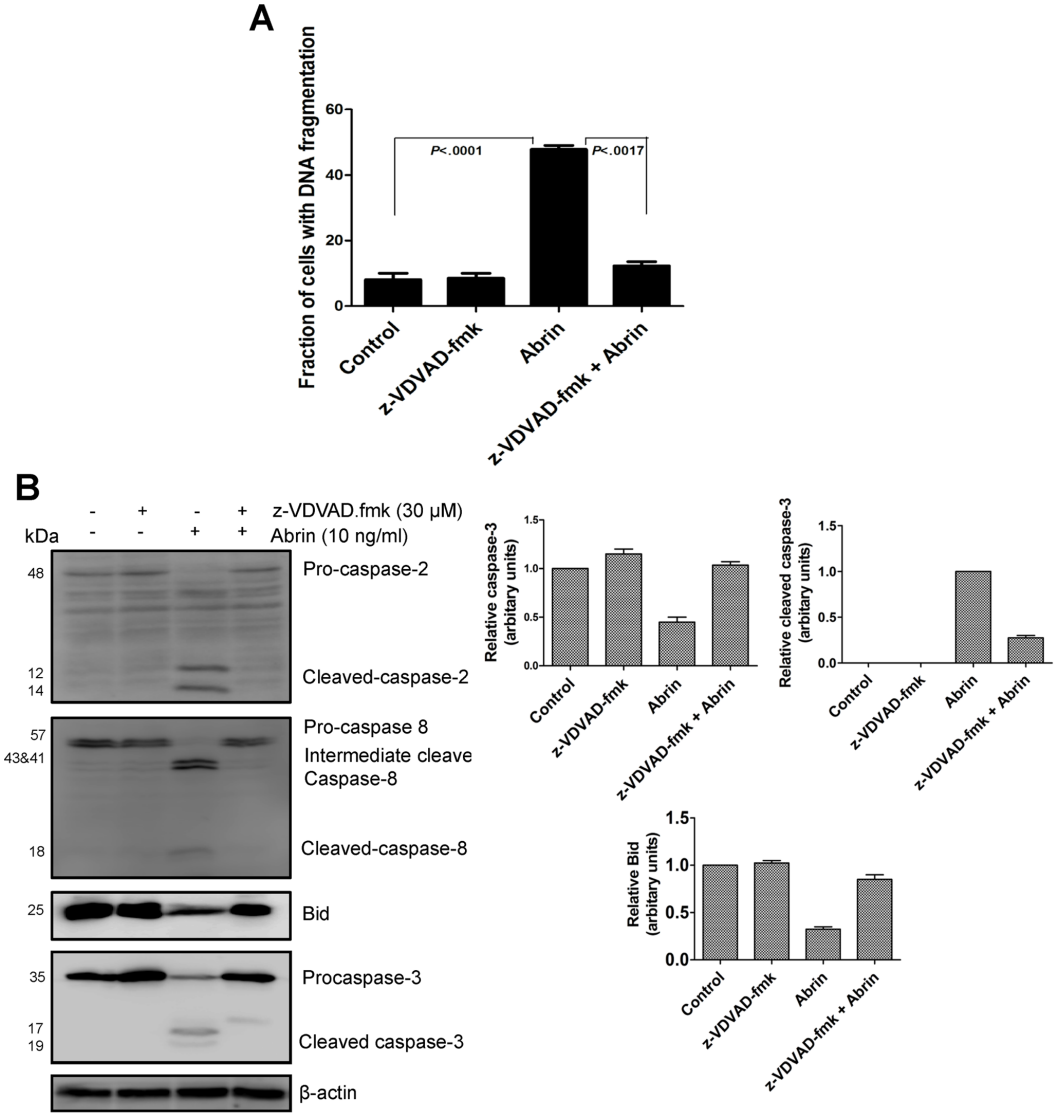
Role of caspase-2 in abrin-induced apoptosis. Jurkat cells were pretreated with caspase-2 inhibitor, z-VDVAD.fmk for 2 h and then with abrin for 10 h. Abrin-induced apoptosis in the presence of inhibitor were quantified by flow cytometry (A) after the treatments cells were harvested, fixed with 70% ethanol, stained with propidium iodide and quantified for apoptotic population. After the similar treatments whole cell lysates were prepared and analysed by Western blot for (B) cleaved caspase-2, 8, 3, and full length Bid. Equal protein loading was checked by stripping and re-probing the membranes for β-actin. Densitometry analysis was performed for two to three Western blots. Each bar is presented as mean ± SE of triplicate samples.

**Figure 5:**
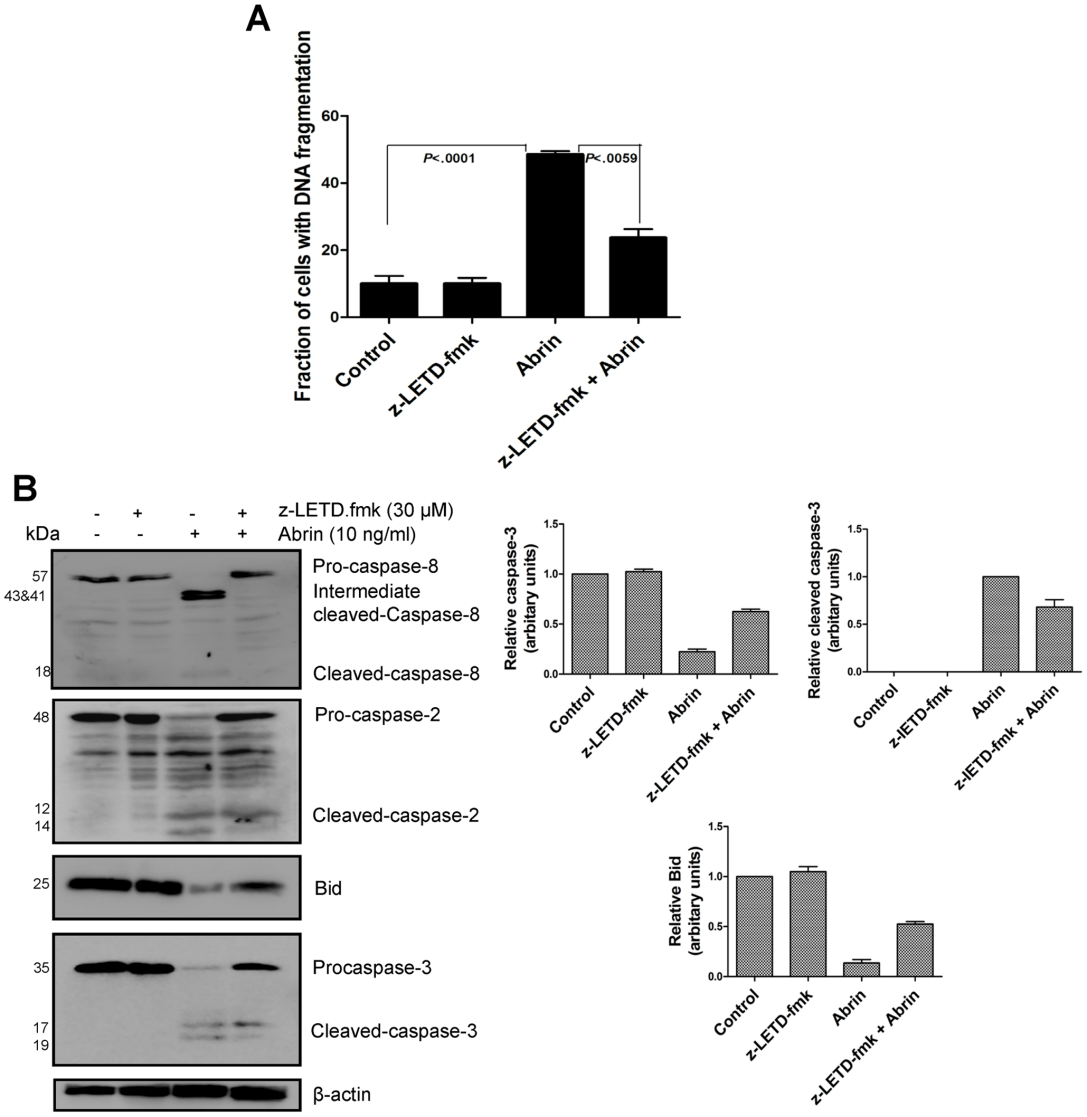
Role of caspase-8 in abrin-induced apoptosis. Jurkat cells were pretreated with caspase-8 inhibitor, z-LETD.fmk for 2 h and then with abrin for 10 h. Abrin-induced apoptosis in the presence of inhibitor was quantified by flow cytometry (A) after the treatments cells were harvested, fixed with 70% ethanol, stained with propidium iodide and quantified for apoptotic population. After the similar treatments whole cell lysates were prepared and analysed by Western blot for (B) cleaved caspase-8, 2, 3, and full length Bid. Equal protein loading was checked by stripping and re-probing the membranes for β-actin. Densitometry analysis was performed for two to three Western blots. Each bar is presented as mean ± SE of triplicate samples.

To understand the importance of activation of caspase-2 and -8, we pretreated Jurkat cells with 30 μM of the cell permeable caspase-2 inhibitor, z-VDVAD.fmk or caspase-8 inhibitior, z-LETD.fmk for 2 h respectively before treatment with abrin (10 ng/ml) for 10 h. The Caspase-2 inhibitor, z-VDVAD.fmk (30 μM) blocked abrin-induced apoptosis by ∼90% (Figure 4A). Caspase-8 inhibition by z-LETD.fmk (30 μM) also decreased abrin-induced apoptosis but the rescue was only about 50% (Figure 5A). These results indicated that abrin-induced apoptosis is dependent on activation of both caspase-2 and -8. Western blot results showed that caspase-2 inhibitor, z-VDVAD.fmk inhibited abrin induced activation of caspase-8 and Bid cleavage (Figure 4B). It also inhibited caspase-3 activation (Figure 4B). However, inhibition of caspase-8 only partially reduced caspase-2 cleavage and showed considerable reversal in cleaved caspase-3, and full length Bid level (Figure 5B). These results suggested that the activation of caspase-2 plays an effective role in induction of apoptosis in Jurkat cells [32].

### The p38 MAPK Pathway is Involved in Abrin-induced Apoptosis

**Figure 6:**
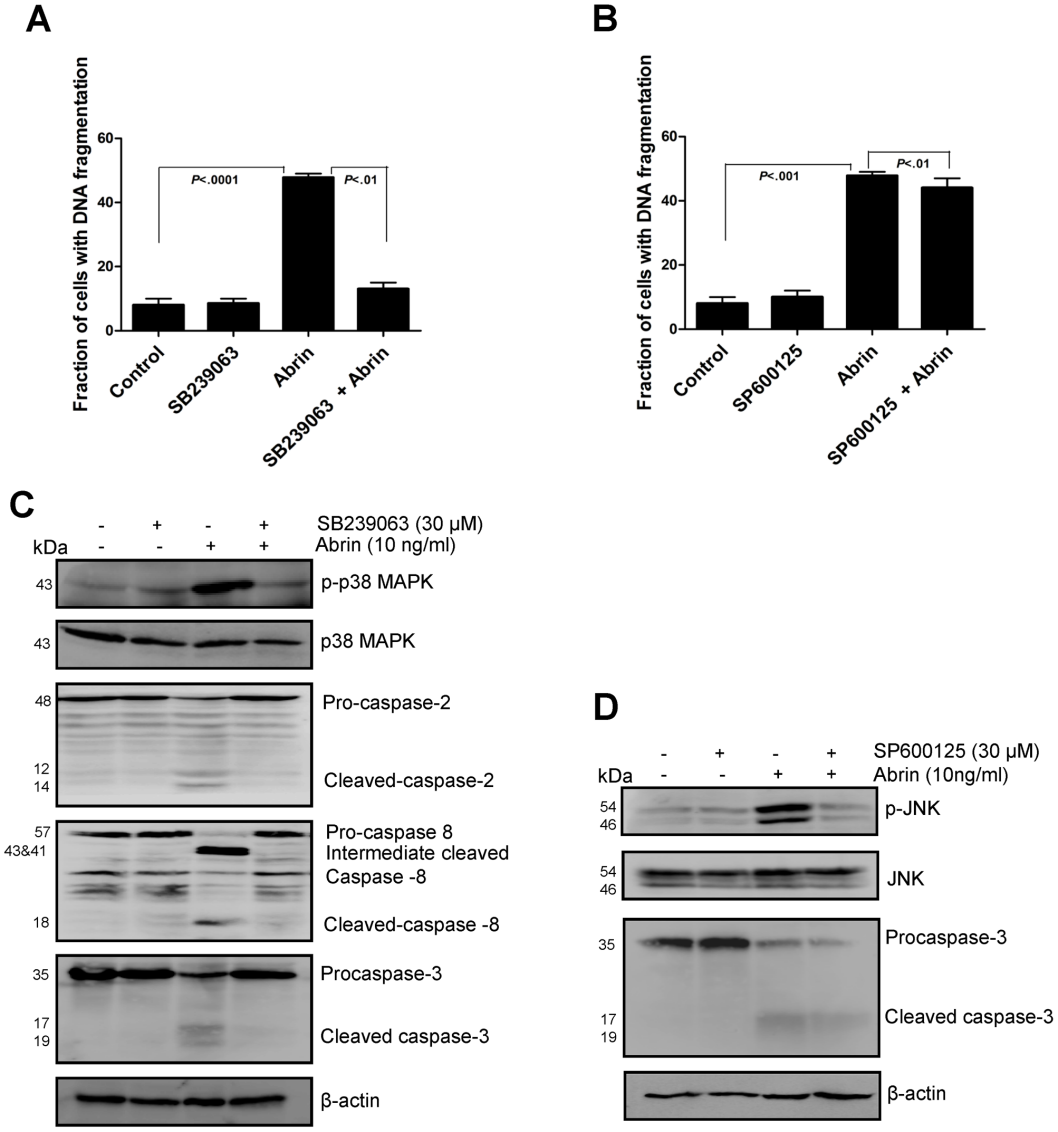
Abrin causes activation of p38 MAPK pathway which leads to apoptosis. Jurkat cells were pretreated for 2(10 ng/ml) for 10 h. (A&B) After the treatments, Cells were harvested fixed with 70% ethanol, stained with propidium iodide and quantified for apoptotic population. Each bar is presented as mean ± SE of triplicate samples. After the similar treatments,whole cell lysates were prepared and analysed by Western blot for (C) p-p38 MAPK, total p38 MAPK caspase-2, 8 and 3. (D) For p-JNK, total JNK and caspase-3. Equal protein loading was checked by stripping and re-probing the membranes for β-actin.

MAPK has been studied in various signal transduction pathways activated by stress [26]. Among the MAPK pathway proteins, the p38 MAPK is shown to have pro-apoptotic effect following ER stress [21], [27]. The involvement of p38 MAPK and JNK pathways in abrin-induced apoptosis was analyzed by using respective inhibitors. The p38 MAPK inhibitor, SB239063 rescued abrin-induced cell death but the JNK inhibitor, SP600125 was found to be ineffective (Figure 6A & 6B). Western blot results showed that p38 MAPK inhibitor, SB239063 which completely blocked phosphorylation of p38 MAPK, also inhibited abrin-caused activation of caspase-2, -8 and -3 (Figure 6C). Moreover, a complete decrease in JNK phosphorylation was observed upon treatment with JNK inhibitor, SP600125 but caspase-3 cleavage was not prevented (Figure 6D). These results suggest that ER stress mediated activation of p38 MAPK and not JNK is involved in activation of caspases during abrin-induced apoptosis.

### Effect of Inhibiton of p38 MAPK, Caspase-2 and Caspase-8 on Abrin-induced Mitochondrial Membrane Potential (MMP) Loss

**Figure 7:**
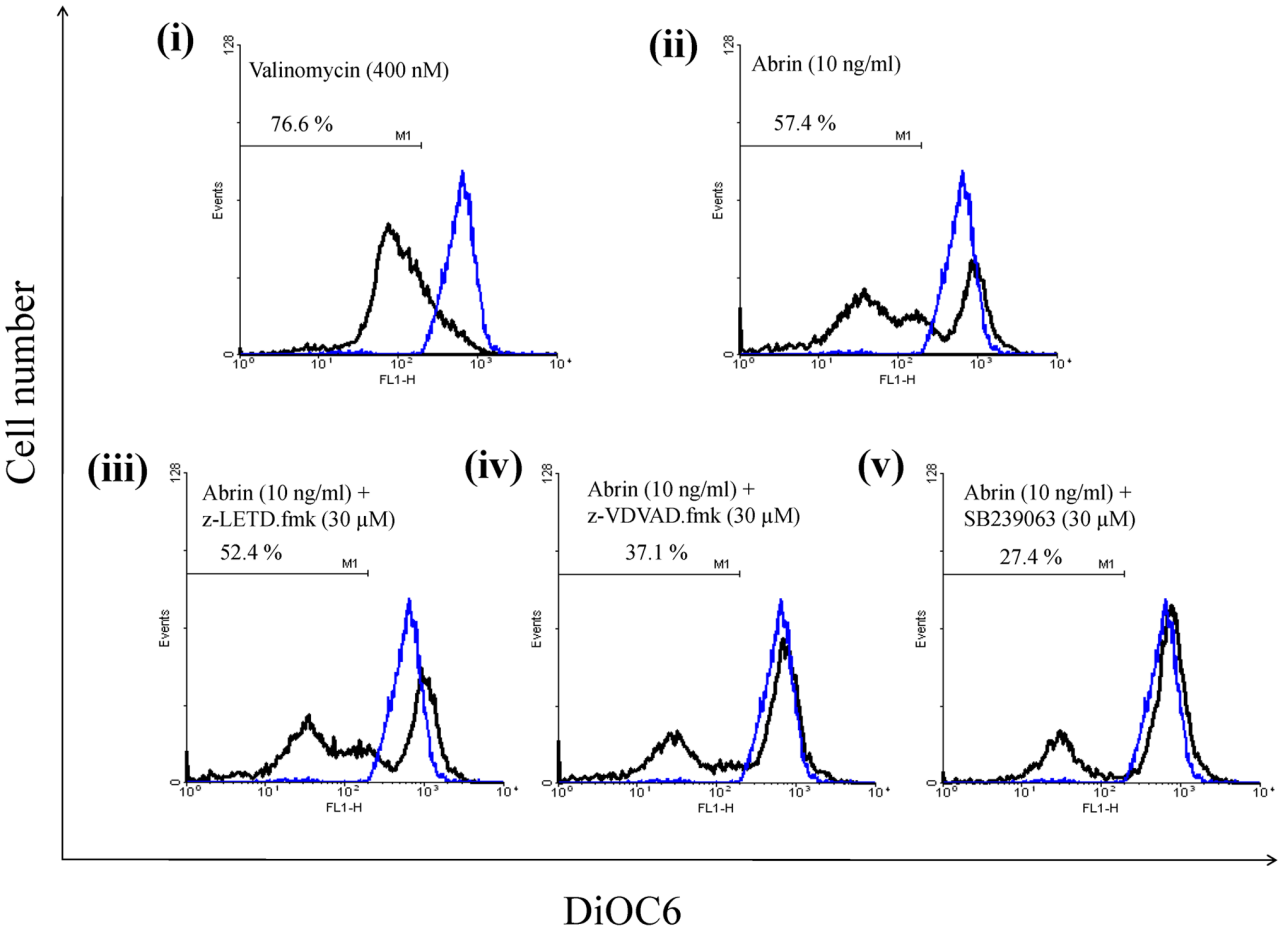
Role of p38MAPK, caspase-2 and caspase-8 on abrin-induced mitochondrial membrane potential (MMP) loss in Jurkat cells. (A) Jurkat cells treated with abrin alone or along with inhibitors were harvested and stained with DiOC6 dye and the percentage of cells positive for green fluorescence were analyzed by flow cytometry as described in Materials and methods. The blue line indicates untreated Jurkat cells and the black line represents Jurkat cells treated as indicated in each panel. Also, percentage in each panel denotes the loss of mitochondrial membrane potential.

Abrin is known to induce apoptosis by disrupting the mitochondrial membrane potential [5]. The factors leading to MMP loss is not well understood. Our studies have demonstrated the involvement of ER stress mediated activation of p38 MAPK, caspase-2, and -8. So, we investigated whether their activation leads to MMP loss. MMP loss by abrin and valinomycin (positive control) was observed by DiOC6 staining. This loss of MMP was significantly inhibited by pretreatment of cells with p38 MAPK inhibitor, SB239063 and caspase-2 inhibitor, z-VDVAD.fmk but not by caspase-8 inhibitor, z-LETD.fmk. (Figure 7A). Overall, we demonstrate the involvement of p38 MAPK and caspase-2 in abrin-mediated MMP loss and finally apoptosis. Since the rescue was not complete, we cannot rule out the activation of multiple signaling pathways.

### Abrin-induced DNA Damage Signalling Pathways

**Figure 8:**
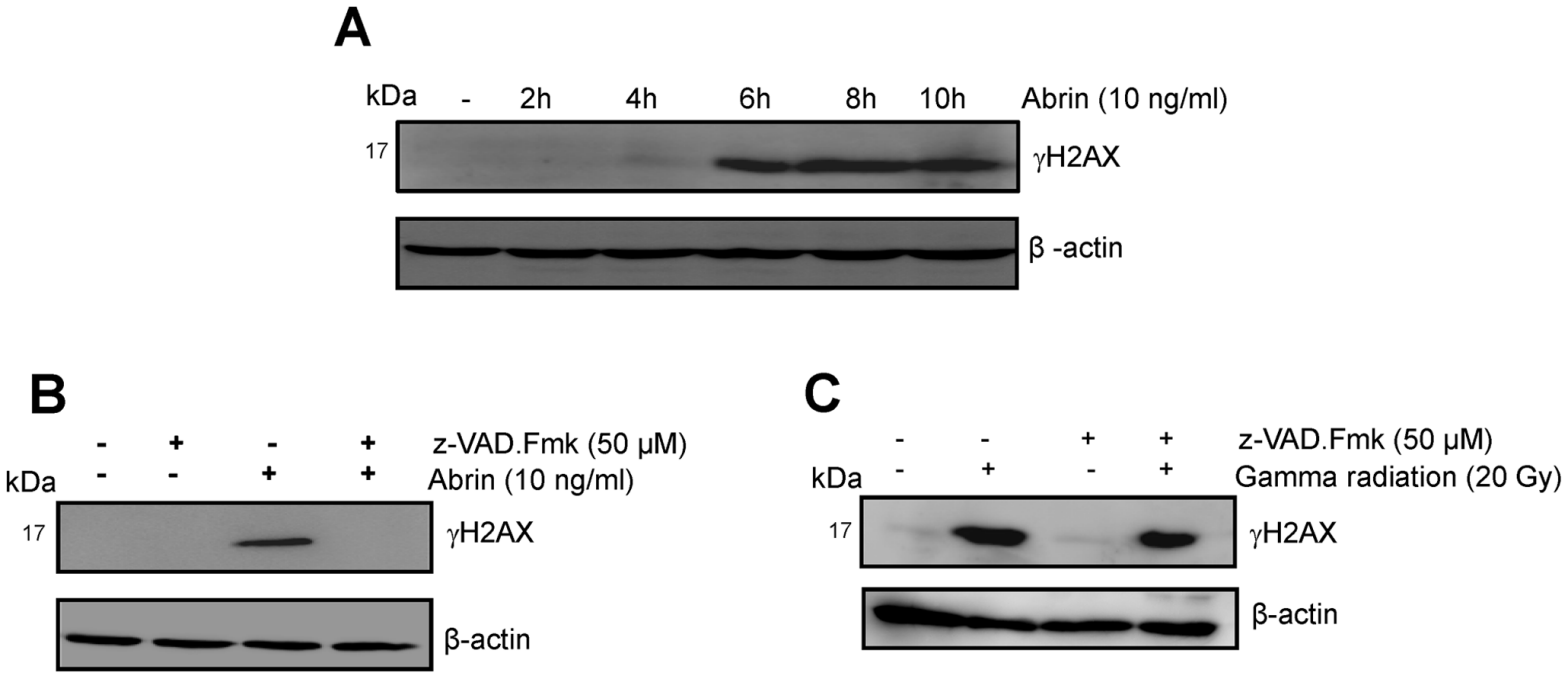
Abrin activates DNA damage signaling pathway. Phosphorylation of H2AX (γH2AX) was analysed in Jurkat cells (A) treated with abrin (10 ng/ml) for different time intervals (0–10 h) (B) pretreated with broad spectrum caspase inhibitor, z-VAD.fmk for 2 h and then with abrin or (C) with gamma radiation (20 Gy). Equal protein loading was checked by stripping and re-probing the membranes for β-actin.

Many type II RIPs have been reported to have nuclease activity and induce DNA damage [22], [23], [28]. In some studies it has been shown that DNA damage signaling pathways can lead to activation of caspase-2 and -8 [29], [30]. We wanted to study whether abrin has a direct role in DNA damage. We checked for phosphorylation of H2AX (γH2AX), marker for double strand DNA break, to determine activation of DNA damage signaling pathway. As can be seen in Figure 8A, abrin induced phosphorylation of H2AX after 6 h of treatment. We assessed whether phosphorylation of H2AX is triggered by the primary DNA damage caused by abrin directly or is a consequence of DNA fragmentation owing to the induction of apoptosis. The phosphorylation of H2AX was analysed in Jurkat cells treated with abrin in presence of the broad spectrum pan-caspase inhibitor (z-VAD.fmk). We found that treatment with abrin in presence of z-VAD.fmk eliminates phosphorylation of H2AX (Figure 8B) but does not affect the induction of phosphorylation of H2AX triggered by ionizing radiation (Figure 8C). Since nuclear ATM is known to co-localize with γH2AX at the site of Double strand breaks in response to DNA damage [41] we analysed the cell lysates also for phosphorylation of ATM (at serine 1981) upon abrin treatment which was completely abolished after pretreatment of Jurkat cells with broad spectrum pan-caspase inhibitor (z-VAD.fmk) as shown in Figure S3. These experiments indicate that DNA damage is a consequence and not the cause of induction of apoptosis upon abrin treatment.

## Discussion

Abrin is a potent toxin belonging to RIP family. It is known to exhibit its toxicity by inhibiting protein synthesis machinery and also by inducing apoptosis in cells. The mechanism by which abrin inhibits protein synthesis is well defined [31]. However, the dependence of apoptosis on inhibition of protein synthesis is not very clear. ER is the major site for protein synthesis, protein folding and secretion as well as calcium storage. Any change in homeostasis of ER can activate a conserved signal transduction pathway known as unfolded protein response (UPR). IRE1, PERK and ATF6 are the three transmembrane proteins which transduce the unfolded protein signal across the ER [7]. Studies on Shiga toxin, a type II RIPs has reported activation of all the three branches of UPR [25] and similar study on type II RIP of plant origin (ricin, riproximin and volkensin) also demonstrated activation of PERK and ATF6 but not the IRE1 pathway [24]. In our study, we observed phosphorylation of eIF2α and upregulation of CHOP indicating that UPR gets activated upon abrin treatment. Our results support earlier observations with other type II RIPs which are known to activate UPR. Apoptosis in response to ER stress has been studied extensively [9]. The stress kinases such as p38 MAPK and JNK have been shown to play an important role in ER stress mediated apoptosis [18]. Also both p38 MAPK and JNK pathway have been implicated in ricin and Shiga toxin induced apoptosis [26], [27], [32], [33]. Both these pathways were found to be activated in Jurkat cells upon abrin treatment but only p38MAPK pathway played a crucial role in abrin-mediated apoptosis.

Earlier studies have also shown that caspase-2 mediated cleavage of Bid can act as a major apoptotic signal downstream of ER stress and thus connects the ER stress to mitochondrial apoptotic machinery. It was found that caspase-2 is localized to ER and activation of caspase-2 preceeds the activation of caspase-3 after ER stress [11]. Caspase-8 has also been shown to participate in ER stress-mediated apoptosis [14], [15]. Studies have shown that the transcription factor CHOP, when activated by ER stress, can increase the expression of cell membrane death receptor DR5 by binding to the CHOP-binding site in 5′-flanking region of the DR5 gene and thus couples ER stress signal to a DR5/caspase-8 related apoptotic cascade [34]. Activation of caspase-8 has been reported before with Shiga and abrin toxin. Shiga toxin activates calpain, a protease which can cause early cleavage of caspase-8 [25] and recently abrin was shown to induce upregulation of Fas ligand and FADD (Fas associated protein with death domain) receptor [35]. In the present study, as abrin is shown to induce ER stress, we explored the involvement of caspase-2 and caspase-8 as key molecules linking ER stress to apoptosis. Activation of both, caspase-2 and -8 was shown in Jurkat cells after abrin treatment and apoptosis was significantly inhibited in the presence of their inhibitors. Caspase -2 played a major role as caspase-2 inhibitor rescued apoptosis caused by abrin to a greater extent than caspase-8 inhibitor. We also observed caspase-mediated activation of the pro-apoptotic protein Bid. Caspase-2 induced apoptosis was shown to involve caspase-8, Bid and caspase-3 cleavage. All these molecules activated by abrin were almost completely inhibited by caspase-2 inhibitor; however caspase-8 inhibitor showed only partial reversal in the level of full length Bid and caspase-3 cleavage suggesting the dominant role of caspase-2 in apoptosis induced by abrin. Also, caspase-2 cleavage was only partially inhibited in the presence of caspase-8 inhibitor suggesting that abrin can activate caspase-2 independent of caspase-8.

The p38 MAPK and caspase-2 inhibitor partially rescued MMP loss upon abrin treatment delineating their involvement upstream of mitochondria. These results also suggest that inhibition of protein synthesis could be the major cause for inducing ER stress leading to activation of p38 MAPK and caspase-2, finally leading to MMP loss. However since rescue of MMP loss is partial, we cannot neglect the possibility that multiple pathways can be triggered by abrin.

Many type II RIPs have been reported to have DNase activity apart from their RNA-N glycosidase action [28]. It has been reported that activation of DNA damage signaling pathway can lead to apoptosis through activation of p53 and caspase-2 [29], [30]. To investigate any direct DNA damage caused by abrin, we checked for the phosphorylation of H2AX which is a marker for double strand DNA breaks [36]. We found significant phosphorylation of H2AX and ATM after abrin treatment which was completely abrogated in the presence of broad spectrum pan-caspase inhibitor. H2AX phosphorylation induced by ionizing radiation was not affected by broad spectrum pan-caspase inhibitor suggesting that H2AX phosphorylation was not a cause but consequence of apoptosis induced by abrin.

To summarize, our studies provide insights into the role of inhibition of protein synthesis towards activating the signaling cascasde upstream of mitochondria in abrin-induced apoptosis. Our findings could be of importance because abrin is extremely lethal and has been classified as a potential biological warfare agent [37]. Yet, there is no antidote or vaccine available against abrin [38]. Inhibitors of many of these elucidated signaling pathways can be considered as an effective antidote against abrin intoxication. Immunotoxins based on abrin are also of therapeutic interest in cancer research [39], [40], [41]. Combined treatment of immunotoxins and ER stress inducers can overload the UPR machinery of cancer cells at concentrations that are well tolerated by normal cells and may show synergistic effect in elimination of cancer cells [24]. Overall, our studies have delineated biochemical mechanisms for apoptosis induction in cells by abrin.

## Materials and Methods

### Purification of Abrin

Using previously standardized protocols abrin was purified from the seeds of *Abrus precatorius* and dissolved in 50 mM phosphate buffer pH 7.4 containing 150 mM NaCl (PBS) and the concentration was determined based on molar absorption co-efficient ε of 100 170 M^−1^cm^−1^ at 280 nm [42], [43]. The purity of abrin preparation was confirmed by coomassie blue staining of SDS-PAGE gel (Figure S1).

### Cell Lines and Reagents

JR4 clone of Jurkat cells (human T-cell lines) were obtained from the National Centre for Cell Sciences (NCCS) and cultured in Roswell Park Memorial Institute (RPMI-1640, Sigma-Aldrich) supplemented with 10% fetal bovine serum (FBS, PAN biotech), 100 I.U./ml penicillin and 100 I.U./ml streptomycin in a humidified atmosphere (5% CO_2_) at 37°C. The cells were passaged every 3–4 days in TC 75 cm^2^ flask. The primary antibody for caspase-2, -3, -8, Bid, eIF2α, JNK, p38 MAPK, phospho-eIF2α, phospho-JNK and phospho-p38 MAPK were purchased from Cell Signalling Technology. Antibodies for γH2AX and P-ATM were from BD Pharmingen. Antibody for β-actin was purchased from Sigma-Aldrich. The Peroxidase conjugated secondary antibodies were from Dako. Propidium iodide (PI) and DiOC6 dye were from Molecular Probes, USA. Annexin-V-FITC apoptosis detection kit I was purchased from BD Biosciences. ECL detection kit, broad spectrum caspase inhibitor z-VAD-fmk, caspase-2 inhibitor z-VDVAD-fmk (Z-Val-Asp(OMe)-Val-Ala-Asp(OMe)-fmk), caspase-8 inhibitor z-LETD-fmk (Z-Leu-Glu(OMe)-Thr-Asp(OMe)-fmk), JNK inhibitor SP600125 and p38MAPK inhibitor SB239063 were purchased from Calbiochem.

### Assay for Protein Synthesis

The assays were carried out in 48 well culture plates. Jurkat cells (0.5×10^6^/ml) were washed with PBS and cultured at 37°C in the absence or in the presence of abrin in 500 μl of leucine-free RPMI 1640 medium for 8 h. At the end of the treatements, cells were pulsed with 0.2 μCi of [3H]-leucine (BRIT, India) for 1 h at 37°C. 100 μg of BSA was added as a carrier and the total protein was precipitated with equal volume of 20% trichloroacetic acid (w/v). The precipitated proteins were then centrifuged at 10,000 rpm for 5 min at 4°C, washed twice with 10% trichloroacetic acid (w/v) and dissolved in 40 μl of 2 M NaOH [44]. The radioactivity incorporated was measured in a liquid scintillation counter (Beckman Coulter Inc., USA).

### Propidium Iodide Staining and Fluorescence Activated Cell Scan (FACS) Analysis

Jurkat cells (0.5×10^6^/ml) were cultured in the presence or absence of varying concentration of abrin (16 nM to 0.016 nM) for 10 h. Cells were then centrifuged at 300× g for 5 min at 4°C and resuspended in 100 μl 50 mM PBS. 1 ml ice cold 70% ethanol was added to the cells and kept at −20°C for 30 min. The cells were centrifuged at 800× g for 5 min at 4°C. After washing once with 50 mM PBS, 100 μl staining solution (20 μg/ml PI, 5 μg RNase in PBS) was added to the cells and incubated for 1 h [45] and analysed by FACScan (Becton and Dickinson ).

### Detection of Apoptosis/Necrosis by Annexin-V-FITC and Propidium Iodide Dual Staining

Jurkat cells (0.5 × 10^6^/500 μl) were cultured in the presence or absence of varying concentration of abrin (16 nM to 0.016 nM) for 10 h. They were then washed twice in PBS by centrifugation at 300× g 5 min at 4°C and resuspended in 200 μl of 1× Annexin-V binding buffer followed by staining with Annexin-V-FITC (3.5 μl) and propidium iodide (5 μl) at 37°C for 15 min in the CO_2_ incubator. The cells were analyzed immediately by flow cytometry for red and green fluorescence using FACScan (Becton and Dickinson).

### Measurement of Loss of Mitochondrial Membrane Potential (MMP) in Cells

Jurkat cells (0.5×10^6^/ml) were cultured in presence of different inhibitors (z-VDVAD-fmk, z-LETD-fmk, SB239063) in combination with abrin or only in the presence of abrin (10 ng/ml) for 10 h. After the treatment cells were washed with PBS and then incubated with 3,3′-dihexyloxacarbocyanine iodide (DiOC6, 40 nM in DMSO) at 37°C in the staining solution for 15 min [46]. Stained cells were washed twice with PBS and analysed by flow cytometry for green fluorescence within 10 min. Valinomycin-treated cells (400 nM for 6 h) were used as positive control.

### Immunoblot Analysis of Cell Lysates

Jurkat cells (3×10^6^/ml) were harvested at 10 h after treatment with abrin. In caspase inhibitor studies, cells were treated with z-VAD-fmk, z-VDVAD-fmk, z-LETD-fmk, SP600125 and SB239063 for 2 h and then with abrin for additional 10 h. Cell lysates were prepared using RIPA buffer [50 mM Tris-HCL (pH 7.5), 50 mM NaF, 10 mM Na_3_VO_4_, Protease inhibitors and Phosphatase inhibitor (Sigma-Aldrich)]. Soluble protein concentration was determined using the Bradford assay (Bio-Rad Laboratories). Protein samples (80 μg) were electrophoresed on SDS-PAGE and transferred onto polyvinylidene fluoride membrane by western blotting. Membranes were blocked with blocking buffer for 1 h at room temperature and probed with primary antibody for desired molecule over night at 4°C followed by peroxidise-conjugated appropriate secondary antibody for 1 h at room temperature. Finally proteins were visualized by ECL detection.

### DNA Damage Induction

Jurkat cells were suspended in magnesium free phosphate buffered saline (PBS) at concentration of (3×10^6^/ml) and irradiated on ice using ^137^Cs source at the indicated dose (dose rate, 2.36 Gy/min).

### Statistical Analysis

Each individual experiment is representative of at least three separate experiments. The error bars represent ± SEM of the experiments. The statistical difference between control and treated samples was carried out using student’s *t*-test. Statistical significance was defined at *p* value <0.05.

## Supporting Information

Figure S1 **Purity of abrin:** abrin was electrophoresed under reducing conditions on a 12.5% polyacrylamide gel and stained by coomassie blue. Lane 1, molecular-mass markers; lane 2, abrin.
(TIFF)

Figure S2 **Flow cytometric analysis of Annexin-V-FITC and propidium iodide staining.** Jurkat Cells (0.5 × 10^6^ cells/500 μl) were treated with varying concentrations of abrin (16 nM (1 μg/ml) to 0.016 nM (1 ng/ml) in serum containing medium for 10 h. Cells were washed in PBS and resuspended in 200 μl of 1× Annexin-V binding buffer followed by staining with Annexin-V-FITC (3.5 μl) and propidium iodide (5 μl) at 37°C for 15 min in the CO_2_ incubator. The cells were then analyzed immediately by flow cytometry for red and green fluorescence using FACScan. Jurkat cells cultured in serum containing medium for 10 h and stained with with only propidium iodide (i); with only Annexin-V-FITC (ii); Jurkat cells without the treatment (iii); treated with 0.016 nM, (iv), 0.16 nM (v), 1.6 nM (vi), 16 nM (vii) of abrin and etoposide (2 μM) as a positive control (viii) for 10 h and double stained.
(TIFF)

Figure S3 **Abrin-induced phosphorylation of ATM.** Phosphorylation of ATM was analysed in Jurkat cells (A) treated with abrin (10 ng/ml) for 10 h (B) pretreated with broad spectrum pan-caspase inhibitor, z-VAD.fmk for 2 h followed by abrin for 10 h. Equal protein loading was checked by stripping and re-probing the membranes for β-actin.
(TIFF)
