# Supplementary figures and images for: Endoplasmic Reticulum Stress-Mediated Activation of p38 MAPK, Caspase-2 and Caspase-8 Leads to Abrin-Induced Apoptosis
Source: PLoS One. 2014 Mar 24;9(3):e92586. doi: 10.1371/journal.pone.0092586 (PMC3963924; doi:10.1371/journal.pone.0092586)

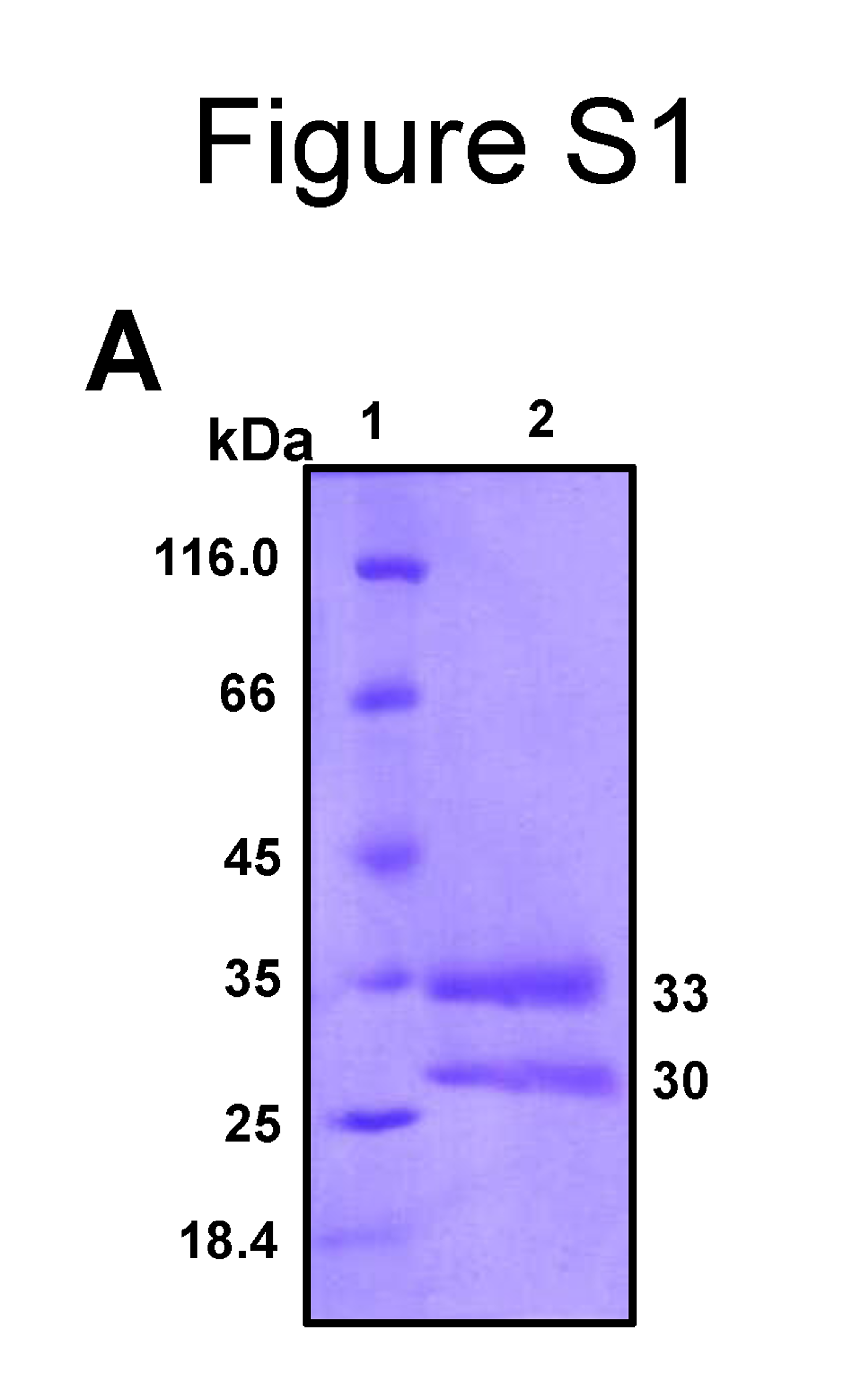

Supplement: Figure S1 — Purity of abrin: abrin was electrophoresed under reducing conditions on a 12.5% polyacrylamide gel and stained by coomassie blue. Lane 1, molecular-mass markers; lane 2, abrin. (TIFF) [file pone.0092586.s001.tif]

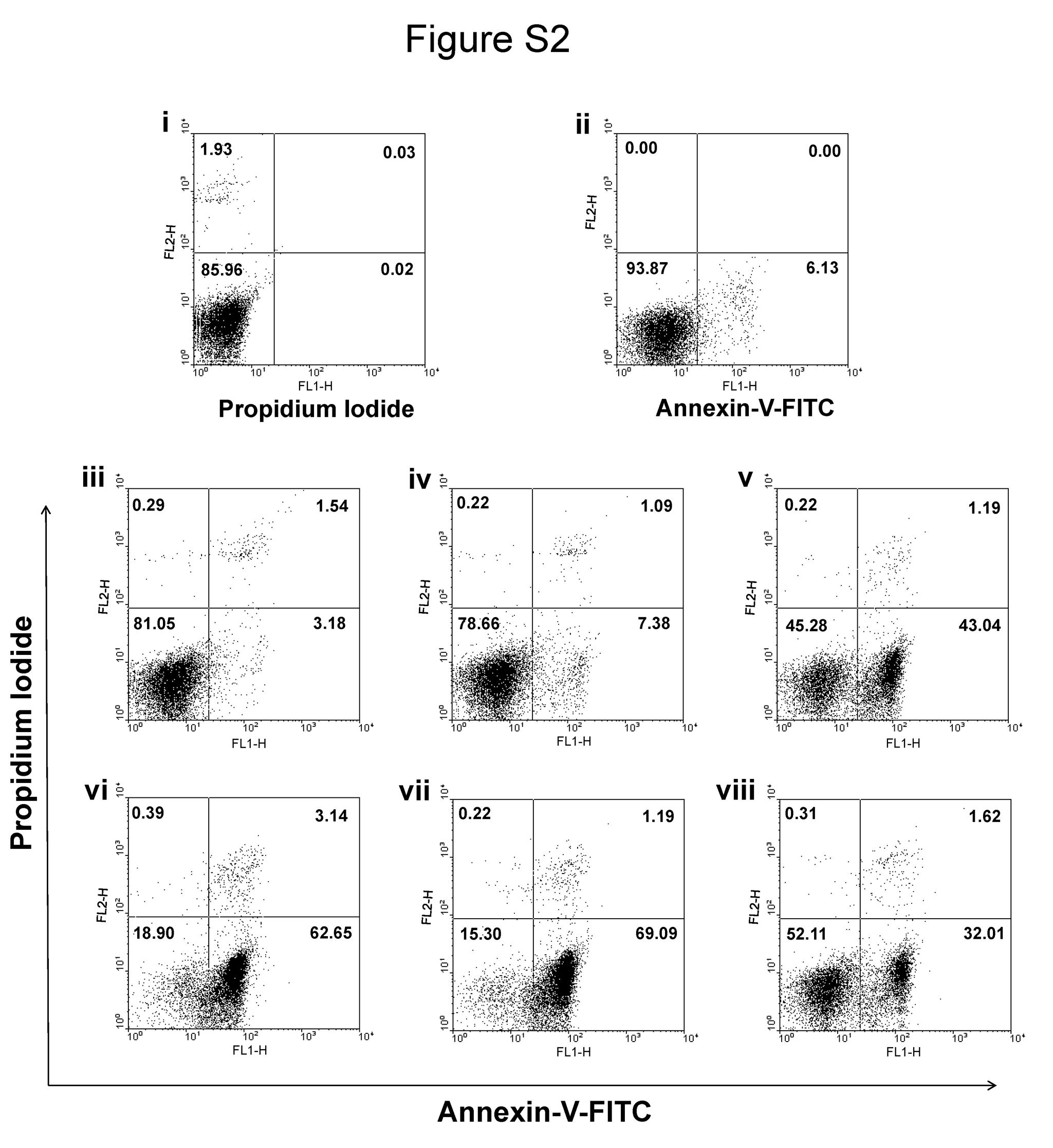

Supplement: Figure S2 — Flow cytometric analysis of Annexin-V-FITC and propidium iodide staining. Jurkat Cells (0.5 × 10^6^ cells/500 μl) were treated with varying concentrations of abrin (16 nM (1 μg/ml) to 0.016 nM (1 ng/ml) in serum containing medium for 10 h. Cells were washed in PBS and resuspended in 200 μl of 1× Annexin-V binding buffer followed by staining with Annexin-V-FITC (3.5 μl) and propidium iodide (5 μl) at 37°C for 15 min in the CO2 incubator. The cells were then analyzed immediately by flow cytometry for red and green fluorescence using FACScan. Jurkat cells cultured in serum containing medium for 10 h and stained with with only propidium iodide (i); with only Annexin-V-FITC (ii); Jurkat cells without the treatment (iii); treated with 0.016 nM, (iv), 0.16 nM (v), 1.6 nM (vi), 16 nM (vii) of abrin and etoposide (2 μM) as a positive control (viii) for 10 h and double stained. (TIFF) [file pone.0092586.s002.tif]

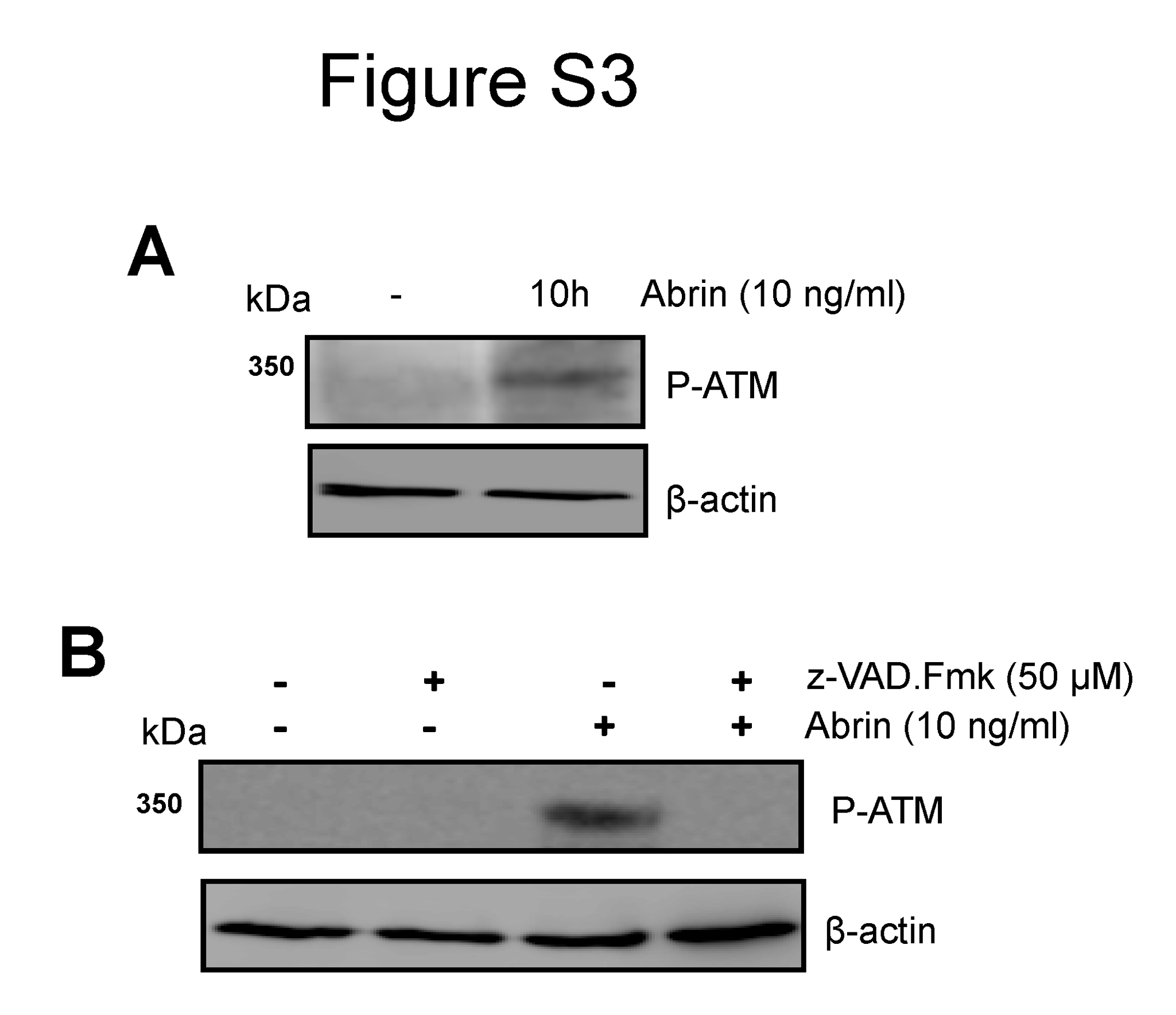

Supplement: Figure S3 — Abrin-induced phosphorylation of ATM. Phosphorylation of ATM was analysed in Jurkat cells (A) treated with abrin (10 ng/ml) for 10 h (B) pretreated with broad spectrum pan-caspase inhibitor, z-VAD.fmk for 2 h followed by abrin for 10 h. Equal protein loading was checked by stripping and re-probing the membranes for β-actin. (TIFF) [file pone.0092586.s003.tif]
